# Online public opinion evaluation through the functional resonance analysis method and deep analysis

**DOI:** 10.1371/journal.pone.0261009

**Published:** 2021-12-31

**Authors:** Linxing Yu, Huaming Chen, Wenqi Luo, Chang Li

**Affiliations:** 1 The College of Literature and Journalism, Sichuan University, Chengdu, Sichuan Province, China; 2 The College of Movie and Media, Sichuan Normal University, Chengdu, Sichuan Province, China; Al Mansour University College-Baghdad-Iraq, IRAQ

## Abstract

A conventional model of public opinion analysis is no longer suitable when the internet is the primary arena of information dissemination. Thus, a more practical approach is urgently needed to deal with this dynamic and complicated phenomenon of propagating public opinion. This paper proposes that the outbreak of internet public opinion and its negative impacts, such as the occurrence of major security incidents, are a result of coupling and the complex interaction of many factors. The Functional Resonance Analysis Method model is composed of those factors and considers the stages of network information dissemination, the unique propagation rule, and textual sentiment resonance on the internet. Moreover, it is the first public opinion governance method that simultaneously highlights the complex system, functional identification, and functional resonance. It suggests a more effective method to shorten the dissipation time of negative public opinion and is a considerable improvement over previous models for risk-prediction. Based on resonance theory and deep learning, this study establishes public opinion resonance functions, which made it possible to analyze public opinion triggers and build a simulation model to explore the patterns of public opinion development through long-term data capture. The simulation results of the Functional Resonance Analysis Method suggest that the resonance in the model is consistent with the evolution of public opinion in real situations and that the components of the resonance of public opinion can be separated into eleven subjective factors and three objective factors. In addition, managing the subjective factors can significantly accelerate the dissipation of negative opinions.

## 1 Introduction

As a barometer of social conditions, public opinion is a crucial element to assist governments to identify social contradictions and maintain social stability. According to the 46th China Statistical Report on Internet Development published by China Internet Network Information Center (CNNIC), China had 940 million netizens, of which 932 million are mobile phone netizens, accounting for 99.2% by September 29, 2020 [1].

The internet has become the main field of engagement for public opinion and is the current information environment that governments, the media, and individuals confront. Significant changes have taken place in the methods of information acquisition and dissemination in the press, television and on the internet. These enable citizens to transcend the limitations of space and time to communicate and to engage in social exchanges with various mobile devices. Hence, a traditional analysis model of public opinion, focusing on simple causality may be unsuitable for the current situation and a more practical method is urgently required.

The development rules of online public opinion are fundamental to good 15 governance [2]. In the network information dissemination environment, the evolution of network public opinion appears disorganized and haphazard [3]. On the contrary, it still presents a certain regularity in the analysis of big data and complex models. To determine the characteristics of network public opinion, many experts and scholars have conducted in-depth discussions and researched this topic from various perspectives. From the perspective of information contagion, the susceptible infected recovered (SIR) model, a classical model of epidemics proposed by Kermack and McKendrick in 1932, [4] and its derivative models, such as susceptible-infected-susceptible (SIS) and susceptible-infected-recovered-susceptible (SIRS), were used to simulate information diffusion among citizens [5, 6]. It should be noted that unlike the spread of viruses, information propagation on the internet does not require communication [7].

As the research in this area progressed and the electronic technology developed, scholars became aware of the existence of these non-contact transmissions affecting online public opinion proliferation. Furthermore, the dynamics of information communication drew their attention [5]. In 2001, the Ising model proposed by Pekalski responded to this consideration. It was applied to analyze stock markets, elections, and other social issues, which indicates that opinion diffusion on a scale-free network is similar to the Gaussian distribution [6, 7]. A discrete mathematical model called Cellular Automata (CA) model establishes a network public opinion incentive model based on CA. When these two theories developed initially, most researchers appreciated their clear individual states and their simple rules. However, these qualities also make these theories incapable of exploring individual interactions in complicated conditions [5]. The voter model, also called the persuasive election model, highlights the impact of the variation in individual opinions on a specific issue on the evolution of network public opinions [8]. The results of individuals’ random interactions with their neighbors, at a particular time, is suitable to explain the opinion change rules in regular networks, but hardly elaborates on the changes on the internet. With the increasing number of internet users, many researchers began to consider the openness and complexity of networks. Based on fuzzy mathematics, a branch of mathematics that studies and focuses on fuzzy phenomena in higher mathematics, Li proposed a fuzzy concept text classification method to construct a fuzzy inference system for emotional expression. It promoted the development of network information monitoring [9]. Meanwhile, Sekhari applied fuzzy sets to the extraction of network public opinion, and constructed a network opinion mining algorithm that was suitable for the analysis of network public opinion in a big data environment [10].

In addition, having realized the importance of emotion in information diffusion on the internet, resonance has gained awareness among scholars. Li’s research pointed out that public opinion resonance is, in essence, emotional resonance. When emotions become universal, they can stimulate common emotion [11].

All the models discussed above present linearity. However, according to the analysis of online public opinion, the factors that influence the quality and efficiency of public opinion include propaganda, dynamics, and emotions. In other words, emergence exists at every stage of public opinion, and its diffusion is no longer confined to the interaction between single factors, but the resonance among multiple factors. To understand the phenomenon mentioned above, this study plans to apply the theory of functional resonance and the method of deep learning to analyze the evolution mechanism of network public opinion. By identifying the functions and the characteristics of network public opinion evolution, this paper presents the functional network diagram and explains the causes of an outbreak of network public opinion, with the aim of proposing a novel method to guide governance of public opinion.

The main contributions of this paper are: First, we proposed the Functional Resonance Analysis Method (FRAM) to analyze and govern online public opinion. In comparison to the traditional model, which focuses merely on the decomposition and the occurrence of public opinion, this model emphasizes its dynamic overall development process from a functional perspective to define its causative factors and the internal evolution mechanism.

Second, based on deep learning, we developed the FRAM model to explain the complexity of public opinion. We used massive data crawled from Sina Weibo and Baidu to train the proposed model; to verify it, we tested it with a real case.

Third, we proposed that the functional resonance triggered by the coupling of functional units was the leading cause of the outbreak of online public opinions. It shows that the identification of critical and non-critical functions is an essential prerequisite for the establishment of a guidance mechanism for public opinion.

Fourth, the FRAM is the first public opinion governance method that simultaneously highlights the complex system, functional identification, and functional resonance. It suggests a more effective method to shorten the dissipation time of negative public 81 opinion and is a considerable improvement over previous risk-prediction models.

The remainder of this paper is organized as follows. Section 2 provides the research related to FRAM, including its definition and history. Section 3 introduces the construction process of FRAM based on deep learning. Meanwhile, section 4 constructs the theoretical model and analyzes its results. Based on this, section 5 presents the simulation process for a realistic case. Finally, section 6 presents the conclusion of this study, highlights some deficiencies, and proposes future research.

## 2 Related works

Functional resonance theory is mainly used for the purpose of accident investigation, 90 safety management, and risk assessment [12]. This theory systematically and fully analyzes all the events and then extracts the crucial elements that lead to dysfunction. Similar to accidents, the outbreak of public opinion is also caused by the interactions of many factors, which provides an opportunity to apply the functional resonance theory to analyze and network public opinion [13].

### 2.1 From classical to functional resonance

Resonance is a classical theory that originates from physics. It has developed from classical resonance and stochastic resonance to functional resonance [14]. Classical (or mechanical) resonance is the most common resonance phenomenon in nature, which means that a system can oscillate with a larger amplitude at some frequencies than at others. A small external force may produce a large vibration, which destroys the phenomenon [15]. A high amplitude vibration in a mechanical or electrical system is caused by a comparatively small intermittent stimulus of the same or similar duration as the normal vibration of the system. Stochastic resonance proposed by physicists Roberto Benzi, Alfonso Sutera andAngeloVulpoiani in 1981 explains more complex systems to solve the problem of Quaternary glaciers, [16, 17]. Unlike classical resonance, stochastic resonance occurs or emerges instantaneously and appears nonlinear. It breaks through the causal relationship to explain how the observed results come into being or emerge from scratch [17].

**Fig 1:**
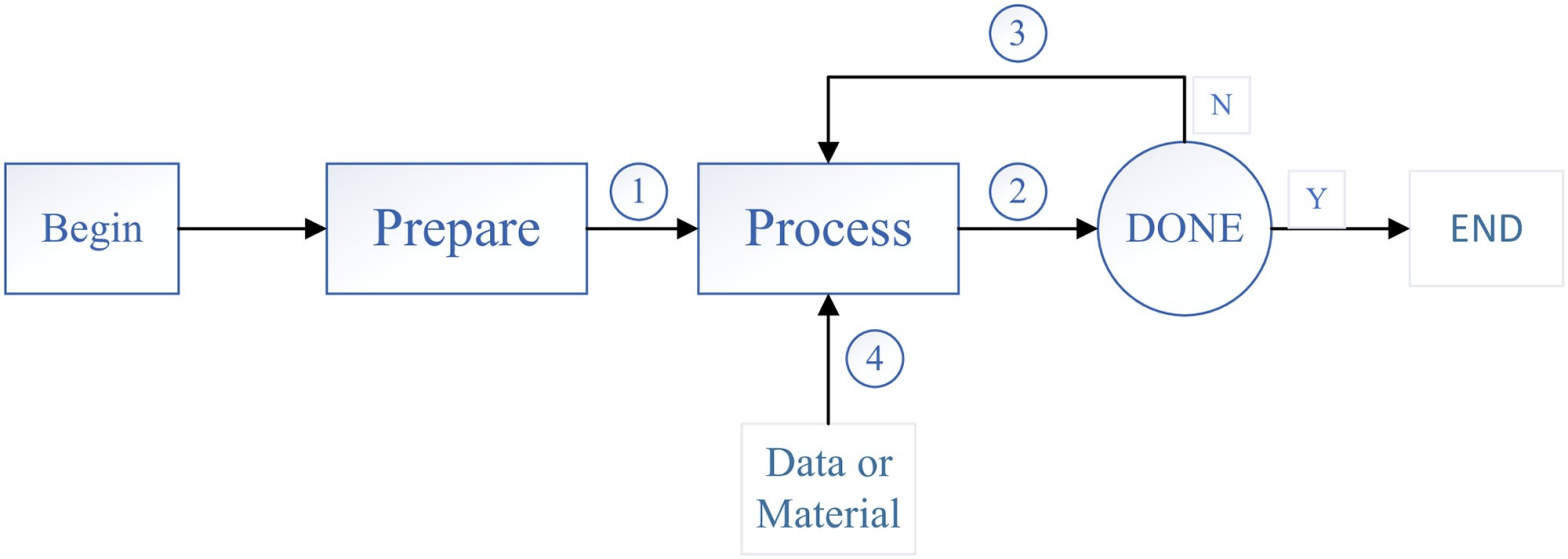
The steps of FRAM.

Functional resonance is an advancement from stochastic resonance, which was first 110 proposed by Professor Erik Hollnagel (Denmark) in 2004. FRAM, a socio-technical system analysis method, analyzes accidents and identifies risk factors in dynamic systems from the perspective of the functional characteristics of the entire system, [12]. Its effects can be seen as a consequence of the system’s functions. Thus, this resonance is called functional resonance rather than stochastic resonance. Functional resonance is both non-causal (emergent) and nonlinear, which impacts the predictability and controllability of the result [17]. The FRAM mainly consists of four steps: identifying and describing functions, identifying changes, judging the aggregation of changes, and analyzing results, as shown in Fig 1.

The first step is to identify the functions required for successful day-to-day work by describing how to do something in detail. These functions form the FRAM model. The second step is to describe the variability of these functions, which incorporates the potential variability of the functions in the model and the expected actual variability of the functions in the model instances. The third step is to analyze the specific instantiations of the model to explain how the variability of the functions may be coupled and to determine if this can lead to unpredictable outcomes. The fourth and final step is to suggest ways to manage potential events of uncontrolled variability in performance that have been identified in the preceding steps.

The links or couplings among the functions are described equally well in the FRAM. There are no arrows, as the FRAM model is a description of the functions rather than a diagram. The sense of the links is not linked to a specific position or direction. While there is a functional tradition of drawing FRAM diagrams, connections may, in theory, be placed anywhere. This is because the functions do not need to be organized or placed in any specific manner in the diagram, for instance, from left-to-right or top-to-bottom.

**Fig 2:**
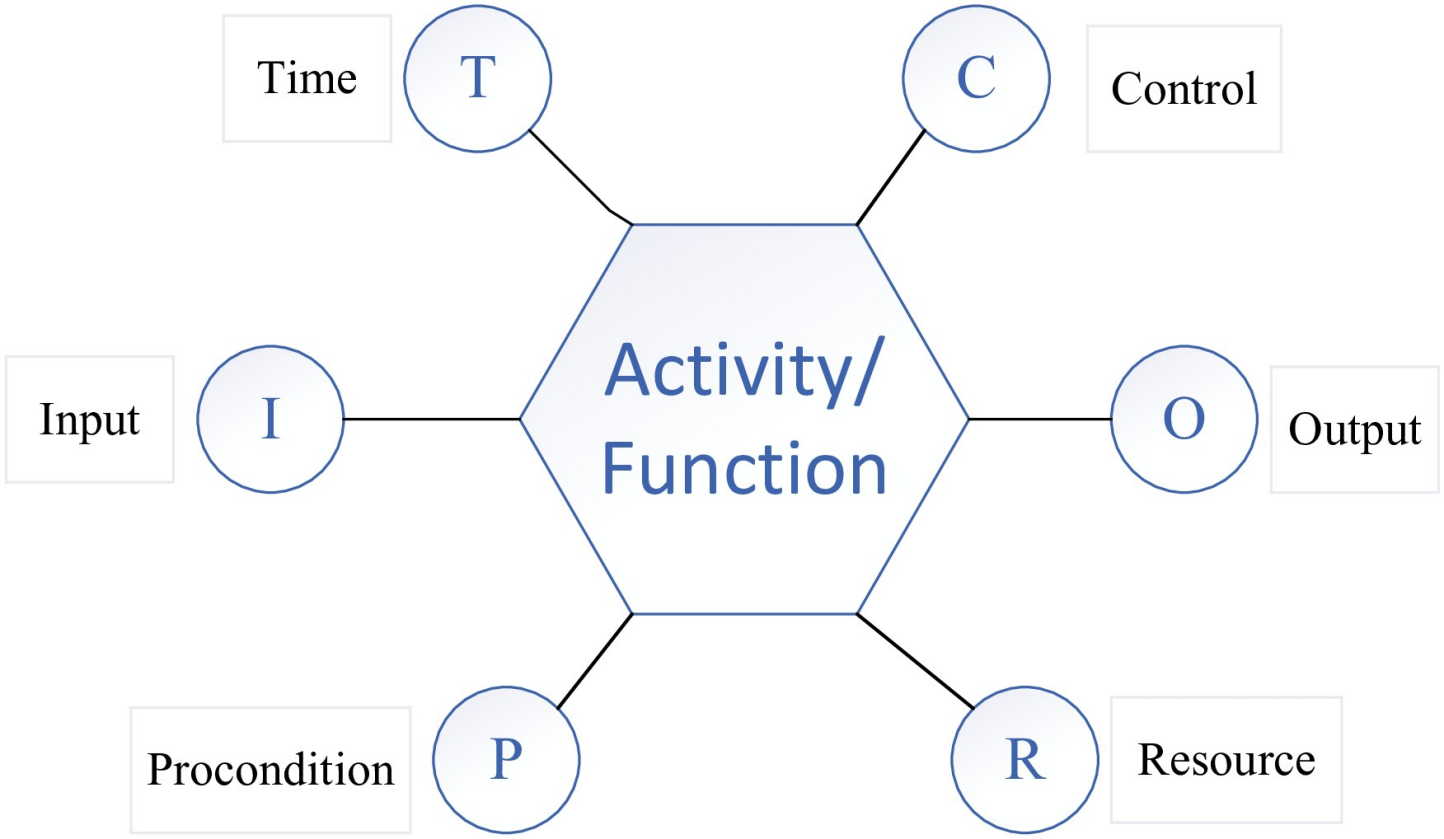
Function identification and description.

The FRAM also clarifies the difference between the functions and how they can be related or coupled. As the first step of FRAM model analysis, function identification and description play an important role in the correct operation mode and function change of the system, as illustrated in Fig 2.

Input (I) refers to the permission or instruction to allow the function to start or do something. Output (O) refers to the result of the function operation, which can be expressed as material, energy, or information, or a system state change. Moreover, preconditions (P) refer to conditions that must be verified before the execution of the function; however, only the input can commence the function. Resources (R) refers to the things needed or consumed for function execution, and can represent material energy, and information technology tools, among others. Meanwhile, time (T) refers to what monitors or adjusts the functions to obtain the expected output. All functions must have control. Control (C) refers to various ways that affect function execution.

### 2.2 Why we use functional resonance to analyze public opinion

Research on public opinion over the internet, demonstrates that public opinion with significant influence and high stimulation to users might not be always be present [18]. However, when a similar event occurs in real life, the memory of the emotion related to this event will be stimulated, and the public opinion event that had dissipated revives to become another heated topic [19]. Especially in the Web 2.0 environment, the dissemination and evolution of netizens’ opinions are more intricate and susceptible. Thus, the evolution of public opinion on the internet is a typical “complex giant system.” Hence, functional resonance theory is a theoretical model based on a complex system. In addition, both functional resonance theory and network public opinion have characteristics of complexity, adaptability and functional coupling.

First, more specifically, complexity constitutes the prerequisite of functional resonance and internet public opinion [20]. In a general information dissemination system, information starts from the initial sender to the receiver and reaches more audience members. As a completely “orderly” dissemination process, the content is constantly adjusted by feedback. However, due to the lack of some functional elements in network public opinion, inaction or improper action causes problems in communication [21]. An element that has an impact on other elements plays a prominent role in the overall system, and eventually leads to an outbreak of online public opinion. In other words, the variation and reorganization of the information dissemination structure leads to the emergence of network public opinion, which conveys the idea of “marking”.

Second, “adaptability” refers to “it can interact with the environment and other subjects” Both functional resonance and internet public opinion follow the law of adaptability [22]. In the theory of functional resonance, the elements of a system are the adaptive subjects of the system. The network public opinion system is also a complex system with many adaptive subjects. The recognition and inclusion of the adaptive subjects provides a useful opportunity for the use of functional resonance theory to analyze network public opinion.

Third, in a complex system, the connection of elements is manifested inside and outside it. In the theory of functional resonance, both the assessment of accidents and the prediction of risks are inseparable from the existence of people, and their interactions or connections are unavoidable. With network public opinion, the outbreak and development are results of the interaction of people. Furthermore, interaction with information, media, and other elements are key to the continuous advancement of network public opinion. Therefore, the application of the functional resonance theory to the governance of network public opinion cannot be separated from the comprehensive consideration of the internal and external complex elements.

Thus, some internal relationships can be found between the two, which implies the possibility of using the functional resonance theory to govern the problem of network public opinion. And the collection method I used complied with the terms of the website.

### 2.3 Resonance of internet public opinion

Presently, there is no consensus on the explanation of the resonance of internet public opinion; however, most scholars agree that the essence of public opinion is the emotional resonance of internet users [23]. In other words, a social event triggers the emotions of some netizens, which infects, spreads and accumulates among netizens, and eventually leads to resonance among netizens [24].

Network public opinion resonance is divided into three categories. The first is the resonance within an event. In this situation, resonance often occurs during an independent network public opinion event. During the development of the event, the public’s emotions and views constantly mix and gradually converge. Moreover, a variety of other factors constantly influence each other to create further resonance.

The second type is the resonance between events. It usually occurs in two or more network public opinion events, which have internal or external similarities, such as the subject or topic. Netizens’ attitudes form a public opinion cluster. When netizens and the media focus on events with the same subject or topic, it causes emotional resonance between events.

The third type is emotion-related event resonance. This often occurs because of the same or similar emotion collection and collision. When netizens experience a certain emotion with an event that arouses their collective memory, this emotion can be amplified, and result in a heated discussion of the event which leads to resonance of emotions. For many network events of this kind, the resonance of emotion-related events are often accompanied by subject-related or topic-related events.

## 3 Construction of functional resonance model based on deep learning

The model in this study was constructed in three steps. The first step was to select the number of popular events from the news in the last 10 years and then obtain the degree of effect of regional and topic factors on the resonance model. The second step was to obtain the attitudes of opinion leaders, netizens, media, government departments and other public opinion bodies about the events, and capture the data of public opinion events via Python 3.7 software from the microblog platform through machine learning (the specific classification code is presented in the appendix). The third step was to verify the simulation model with a realistic case that occurred in 2019.

To ensure the results are reliable, this study adopts long-term and large-scale machine learning; hence, there is no need to predict the influence coefficient of each key element of public opinion. Instead, we obtain the basic rules of public opinion evolution through big data learning, and simulate the governance effect of the functional resonance model based on these rules and preview the occurrence of various situations.

With an aim of knowing the popularity of public opinion events, we calculated the users logging into Webio data or Baidu, and calculated the data of users logged into Weibo. First, we analyzed the mobile data terminal through the Ajax response and then analyzed the data in the JSON format. Then, we filtered out the core events. We used keywords. Finally, after the abnormal data were deleted, samples of mainstream public opinion events were sampled through random sampling. The specific code is provided in the appendix.

**Fig 3:**
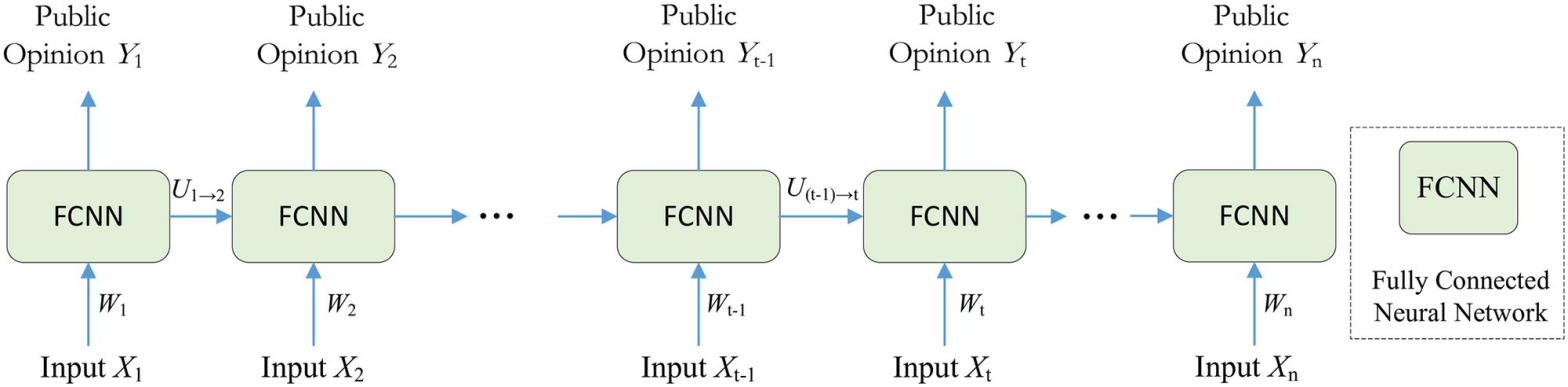
Construction of cyclic, fully connected neural network model.

Based on the evolution of the above model rules, the resonance process and governance effect of public opinion events were simulated. The machine learning algorithm is a process or set of rules to follow in calculations or other problem-solving operations that can automatically analyze and learn rules from data and can be used to predict unknown data. In this study, researchers regard the dissemination and diffusion of information as a neural network system, and use recurrent fuzzy cellular neural network (RFCNN) to perform machine learning under supervision. The process is illustrated in Fig 3.

The simulation model is shown in Fig 3. We use RFCNN to mine, and simulate the relationship between multi-time node input {*X*_1_, *X*_2_, ⋯, *X*_*n*_} and multi time node output {*Y*_1_, *Y*_2_, ⋯, *Y*_*n*_}. In each time node, the hidden unit of RFCNN is taken out and copied. Each copy will transfer the hidden layer value at time *T* − 1 to time *T* through an independent transfer layer. Finally, we initialize the weights randomly, and then optimize the weights by back propagation in the hidden layer. The process of forward propagation in RFCNN is as follows,

$$H_{t}=W_{t}*X_{t}+B_{t}+U_{(t-1)\to t}*H_{t-1}$$ (1)

$$Y_{t}=\sigma (H_{t})=\frac{1}{1+e^{-H_{t}}}$$ (2)

where *W*_*t*_ is the weight parameter of the fully connected neural network (FCNN) at the time *t*.*X*_*t*_ is the input variable of the system at time *t* and the bias of the time fully connected neural network. *B*_*t*_ is the bias of the fully connected neural network at time *t*. *U*_(*t*−1)→*t*_ is the weight of transferring the hidden layer neuron value at time *t* − 1 to time t. *σ* refers to the activation function, and *Y*_*t*_ is the output public opinion value at time *t*.

**Fig 4:**
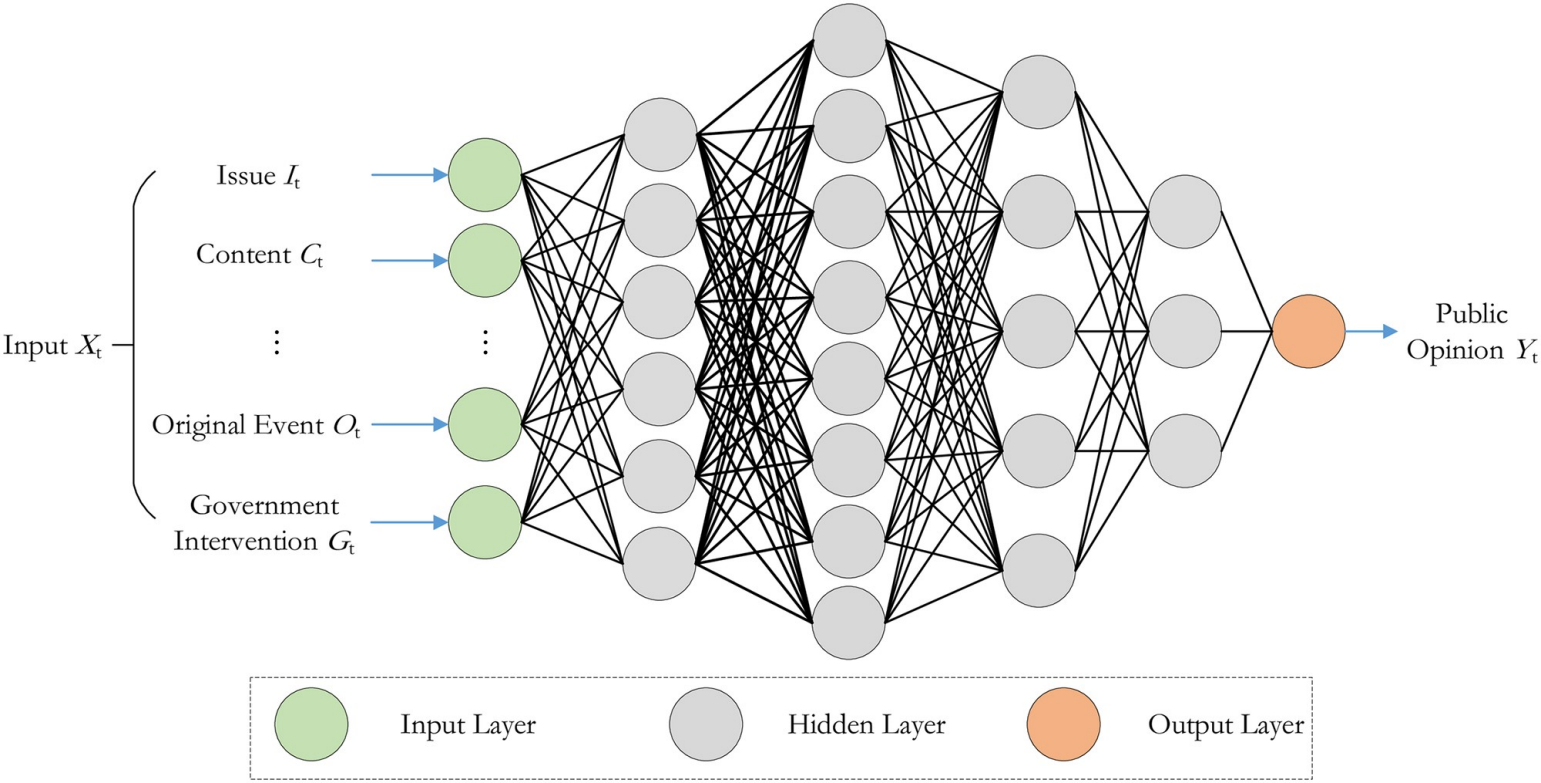
Construction of fully connected neural network model.

According to the description of the model construction scheme described above, it is obvious that the public opinion calculation at each moment is performed by the FCNN. The model structure is illustrated in Fig 4.

**Fig 5:**
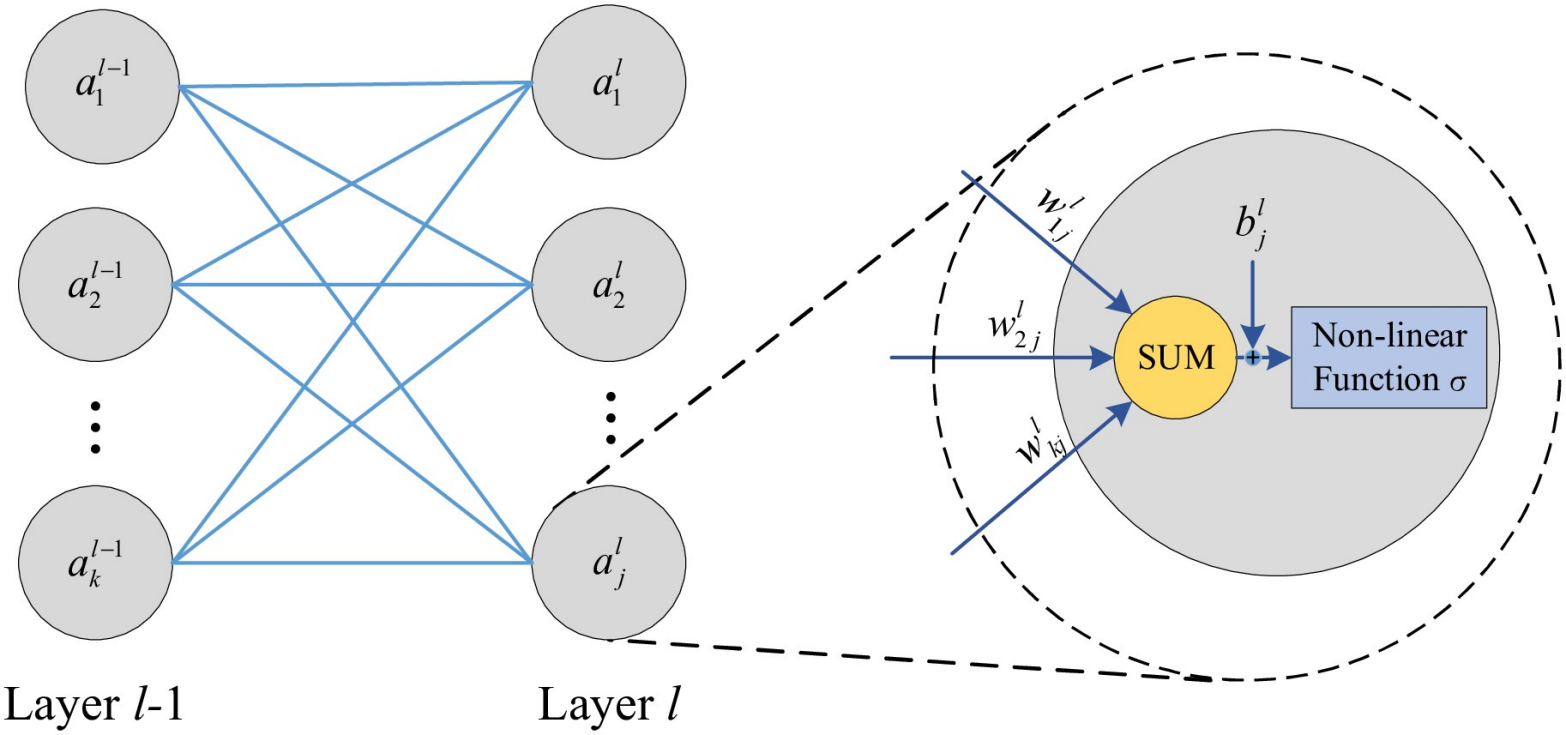
Operation flow chart of adjacent two layers of fully connected neural network.

The FCNN is composed of input layer *X*_*t*_ = {*I*_*t*_, *C*_*t*_, …*O*_*t*_, *G*_*t*_}, output layer *Y*_*t*_, and several hidden layers *s*. The input layer also introduces the output layer *Y*_*t*−1_ of the previous time as the input, which is consistent with the input(I) and output(O) of each functional unit in the functional resonance model. Each neuron (hidden layer) is connected to the output of all previous neurons, and then multiplied by a separate weight, summed, and output to the neurons through nonlinear function processing. It can be observed in Fig 4. that the structure of the FCNN we use is complex and the weight parameters are varied, so it is difficult to express it completely using the formulas. Therefore, we introduce the most basic model example to illustrate the working process of the model. The basic model is shown in Fig 5.

The forward propagation formula of a neural network is as follows. Among them, $z_{j}^{l}$ is the *j*-th neuron of the layer $l,w_{kj}^{l}$ is the weight coefficient of the neuron from *k*-th of the layer *l* − 1 to the *j*-th of the layer; $a_{k}^{l-1}$ refers to the neuron, which is was activated by the *k*-th activation function of the layer $l-1,b_{j}^{l}$ refers to the bias that assigned value to the *j*-th neuron of the layer *l*. It is observable from the above formula that the transfer between layers is obtained by the weighted summation of dense neurons. Until now, all operations have been linear. Before inputting to the next layer, the neurons in the current layer must use a nonlinear activation function to enhance the fit of the model. The following expression represents the activation of the current neuron,

Among them, $z_{j}^{l}$ is the *j*-th neuron of the layer *l*, $w_{kj}^{l}$ is the weight coefficient of the neuron from *k*-th of the layer *l* − 1 to the *j*-th of the layer; $a_{k}^{l-1}$ refers to the neuron which was activated by the *k*-th activation function of the layer *l* − 1, $b_{j}^{l}$ refers to the bias that assigned value to the *j*-th neuron of the layer *l*. It can be seen from the above formula that the transfer between layers is obtained by weighted summation of dense neurons. Up to now, all operations are linear.

Before inputting to the next layer, the neurons in the current layer need to go through the nonlinear activation function to enhance the fitting ability of the model. The following expression represents the activation of the current neuron,

$$a_{j}^{l}=\sigma (z_{j}^{l})=\frac{1}{1+e^{-z_{j}^{l}}}$$ (3)

The above is the operation to obtain the neuron value of the current layer *l*. This “linear + nonlinear” operation is developed layer by layer. When the number of model layers and the complexity is sufficient, any function can be fit to realize mapping between data. For the change of parameters in the neural network, the loss function was used to measure the distance between the output of the current model and the target output. The formula is:

$$L=\frac{1}{2I}\sum_{i}\|y_{i}-p_{i}{\|}^{2}$$ (4)

where *I* represents the number of neurons in the output layer, and *y* and *pi* represent the value of the neuron of the i -th tag in the i -th output.

When updating the weights, the gradient descent algorithm was used. For the weights $w_{kj}^{l}$ from the *k*-th neuron in *l* − 1 layer to the *k*-th neuron in *l* layer, the following formula is used to alternate them:

$$w_{kj}^{l}=w_{kj}^{l}-\eta \frac{\partial L}{\partial w_{kj}^{l}}$$ (5)

For the bias values $b_{j}^{l}$ of the *j*-th neuron in layer *l*, the following formula is used to alternate them:

$$b_{j}^{l}=b_{i}^{l}-\eta \frac{\partial L}{\partial b_{i}^{l}}$$ (6)

## 4 Internet public opinion governance based on FRAM

As the FRAM is a method rather than a mathematical model, it makes no assumptions about how the system under investigation is structured or organized, nor does it address possible causes and cause-effect relations. FRAM is a theory and not a mathematical model. It does not require human predictions about the structure or organization of the system under investigation, nor does it require humans to set out the key elements for opinion generation. Rather than seeking faults and malfunctions, FRAM explains opinion (the cause of emergence) in terms of how functions are coupled and how changes in day-to-day performance trigger resonance. First, we organize the constituent elements of network public opinion, the different performances, and the role of each constituent element in the universal rules of network public opinion. Second, to build the functional resonance model of network public opinion, each function ought to be defined in the model.

### 4.1 Function identification

According to previous academic analysis of the influencing factors of internet public opinion, this study divides the influencing factors into two categories: objective and subjective factors. Among them, the objective factors refer to the social and economic factors that do not change with the development of events. The subjective factors refer to the participants in the public opinion event, which affects the trend in public opinion because of the different performances.

There are 3 objective factors. First, is issue: Internet users give different attention to different social events. It is obvious that topics involving official corruption, social justice, environmental pollution, and religious affairs are highly sensitive and can easily shape public opinion. Second, is region: China has a wide range of regions, and the regional population distribution and economic development are unbalanced. The connectivity, timeliness, and widespread use of internet technology make it possible for everyone in any region to access, spread, and edit information equally. However, in the economically developed and densely populated eastern region, people are more likely to participate in the discussion of network events. Third, is the influence of original events: If the original event attracts the attention of many internet users, media reports, and does not receive a timely response from the government, it may escalate into a large-scale public opinion event.

There are 11 subjective factors. This paper describes the subjective factors of the evolution of network public opinion and determines 11 functions of network public opinion from its generation to its extinction. The functions related to internet public opinion at its beginning are shown in Table 1. Functions of the development of internet public opinion are illustrated in Table 2. Moreover, the functions of the dissipation of internet public opinion are demonstrated in Table 3.

**Table 1:** The functions in the beginning.

	F1 Client & stakeholder post	F2 Opinion leaders communication	F3 Netizens’ attention and participation	F4 Investigation and report of official media
P	Social interests and emotions, Pluralistic values, Lack of truth	Special identity	Interest demands, Tendentious views	Event overview, Interest demands, Tendentious views, Asking for the truth, Views and emotions
I	The incident brock out	Event overview, Interest demands	Event overview, Interest demands, Tendentious views, Asking for the truth	Verification, interview and investigation of the incident
O	Event overview, Interest demands	Tendentious views, Asking for the truth	Views and emotions	News and commentary
C	Delete or paste the cover post	Delete or paste the cover post	Delete or paste the cover post	Internal-internal regulation of media organizations; External: external pressure (politics, law, peers)
R	Social media	Social media	Social media	The Internet
T	The event happened	The event happened	The event happened	Reporting time

**Table 2:** The functions in the development.

	F5 Processing and communication of Internet marketer	F6 Focus and discussion of netizens	F7 Government intervention
P	Purposeful guidance of public opinion	Event overview, Interest demands, Tendentious views, Asking for the truth, Views and emotions, News and commentary, Popular language	The credibility, execution and coordination of the government
I	Event overview, Interest demands, Tendentious views, Asking for the truth, Views and emotions, News and commentary	Emotion and Request	Event overview, Interest demands, Tendentious views, Asking for the truth, Views and emotions, News and commentary, Popular language, Emotion and Request
O	Popular views	Group view and emotion	Supervision and accountability of the units involved, to disclose the progress or truth of an investigation
C	Delete or paste the cover post	Delete or paste the cover post	Internal-internal regulation of media organizations; external: external pressure (politics, law, peers)
R	Social media	Social media	Staff and media
T	Event Report	Event Report	Event Report

**Table 3:** The functions in the dissipation.

	F8 The units involved come forward to solve the problem	F9 Client & stakeholder response	F10 Following reports by official media	F11 netizens’ dissipation and transfer
P	Event overview, Interest demands, Tendentious views, Supervision and accountability of the units involved, to disclose the progress or truth of an investigation	The settlement of events and interest demands	The settlement of events and interest demands	Make public the progress or truth of the investigation, The process and results of the incident investigation, Response to the outcome of the event resolution, Release of the latest results, Leading comments published
I	Investigate the truth, Appease the victim, Punish the relevant personnel	Supervision and accountability of the units involved, Make public the progress or truth of the investigation, Process and result of incident investigation	The process and results of the incident investigation, Response to event resolution results	Attitude and emotion
O	Process and result of incident investigation	Response to event resolution results	Publication of the latest results, Publication of guiding comments	Views and opinions
C	Internal-internal regulation of media organizations; External: external pressure (politics, law, peers)	Delete or paste the cover post	Internal-internal regulation of media organizations; External: external pressure (politics, law, peers)	Delete or paste the cover post
R	Staff and media	Social media	The Internet	Social media
T	Event resolution	Event resolution	Event resolution	Event resolution

**Fig 6:**
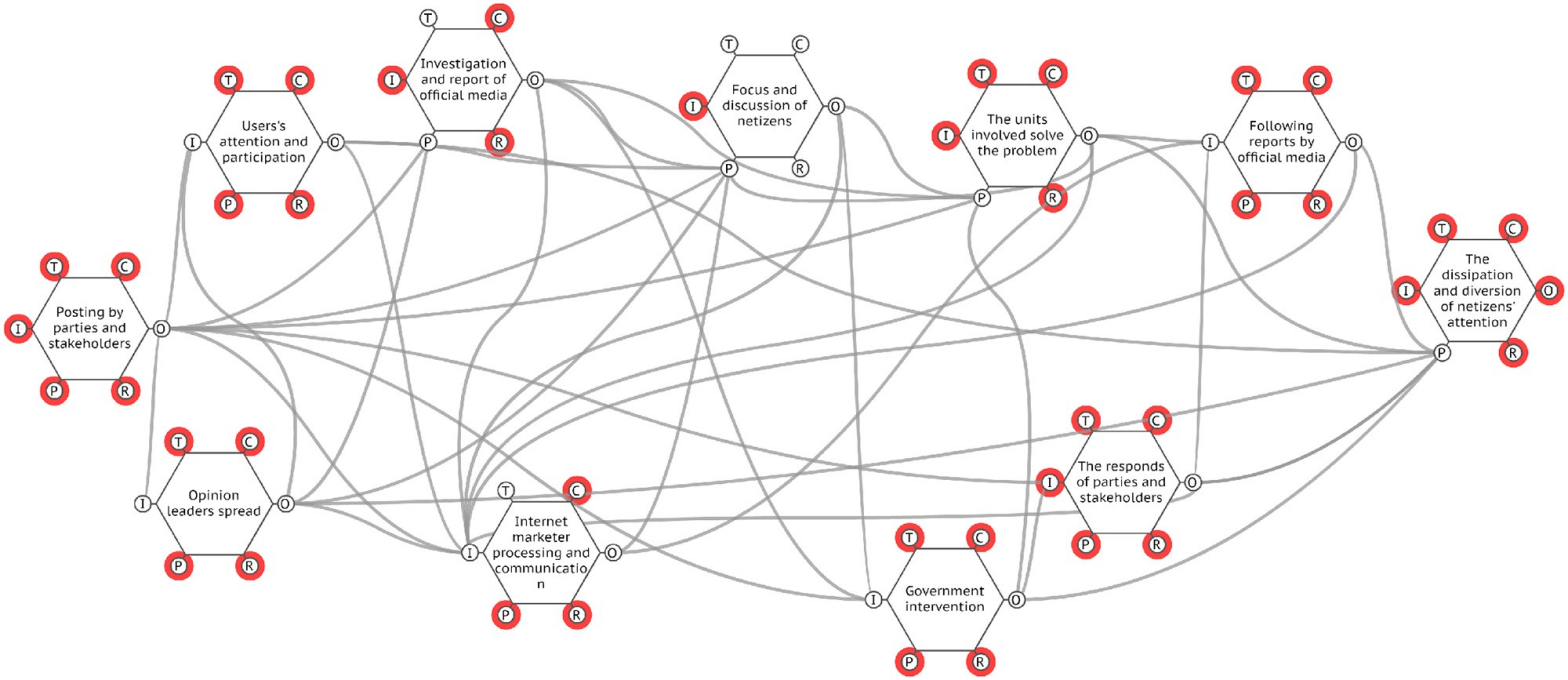
Functional resonance of network public opinion.

According to the above analysis of the functional hexagonal model, we input the six feature descriptions of each function into the FMV (FRAM model visualizer) visualization tool, and then obtained the functional network diagram of the evolution of network public opinion, as shown in Fig 6.

If the functions of the network public opinion system run normally, there will be no functional changes or functional failures. Then, in the entire public opinion system, from public opinion generation to its extinction, risk accidents will not occur. However, the operation of each function in a public opinion system is not immutable in reality. The operational environment of the system and the operation state of the upstream functions affect the normal operation of the downstream function. Therefore, the key to analyzing functional resonance is to identify the changes in key functions and their impact on the operation process of the network public opinion system.

### 4.2 Function labeling

Artificial recognition was used to score the functional elements. Regarding objective factors:

Issue type: Regarding the topic type for social events, we cannot completely determine which topic is more likely to attract public attention. Therefore, when establishing the variables, it is necessary to narrow the value gap. Establishing variables such as 1, 2, 3, and 4 can reduce the differences in the values of variables, and only reflect the type of the variable.

Effect: This is about the influence of the original event. From the data captured on the internet, most personal events did not have a great impact on public opinion. According to the scale of influence, we set regional events to 5 and social events to 10.

Region: This refers to the place where social events occurred. There are several indicators to measure whether the economy is developed, and air quality data can indirectly reflect regional economic development. Therefore, to judge whether it is a developed area, this paper uses the level of the monitored city in the “2019 Bulletin of China’s Ecological Environment” as the measurement standard.

In this study, the 11 main factors are summarized as netizen behavior, opinion leader communication, media reports, and government responses. Netizen behavior refers to information dissemination behavior of ordinary netizens. Because of the substantial number of netizens and the large number of comment platforms, it is unrealistic to fully grasp public opinion. Moreover, the rationality of netizens is determined by statisticians after sampling and artificial evaluation, which is subjective. Therefore, it is not appropriate to set too large a value. Therefore, we set a discrete integer of 1–5, because irrational speech often promotes the development of internet discussion.

Opinion leader communication refers to the authoritative netizens in the field of network communication, whose release of news related to public opinion will change the attitude of most netizens and guide the direction of public opinion. According to the number of fans and the interaction frequency of the fans, an influence value of 13 is obtained. The greater the influence, the greater the value. The objective description weight was set to 1, the positive guidance weight was 1.5, and the negative guidance weight was 2. The public opinion itself is affected by emotion, and the weight of negative emotion is greater than that of positive emotion.

Media reports can arouse netizens’ attention to social events, promote the development of public opinion events, dispel netizens’ negative emotions, and correctly guide the direction of public opinion. Similar to the guiding principle of opinion leaders, the value of guidance with emotion is greater than that of guidance without emotion, and the value of negative emotion is greater than that of positive emotion. Because the direction of public opinion in the media is for all members of society, this factor should occupy a higher position than opinion leaders.

Government response is the timely response and reasonable handling of the incident by the government or relevant parties. These actions can relieve the negative emotions of netizens to the greatest extent. The government’s response is official and authoritative, and often expresses its attitude toward events from the perspectives of objectivity, fairness, and rationality. Moreover, due to the functions of government, the speeches are based on correctly guiding public opinion.

## 5 Simulation experiments

**Fig 7:**
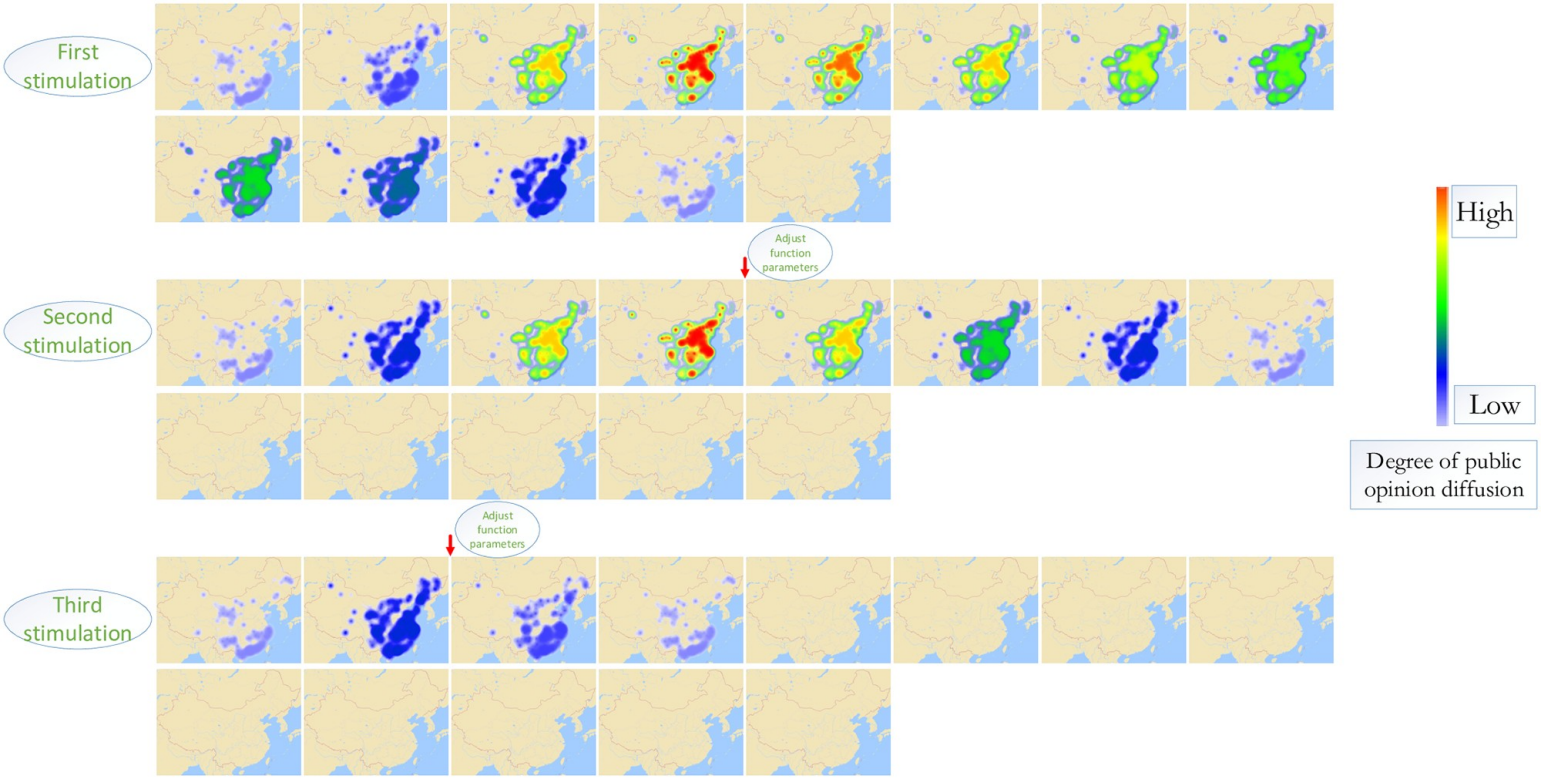
First simulation [25]. Republished from [https://unsplash.com/s/photos/china-map] under a CC BY license, with permission from [Unsplash], original copyright [original copyright 2020].

After the text is modeled by the above operations, the computer can recognize the resonance among functions and stimulate the development of network public opinion. Moreover, to verify the effectiveness of the simulation model, this study selects the “food safety incident of Chengdu No. 7 Middle School” which happened in Chengdu on March 12, 2019, as the study object. The simulation results make the model more convincing in its ability to analyze the evolution of public opinion and to participate in public opinion governance. Through the analysis of the data text, the study represented the development of this event, as shown in Fig 7.

**Fig 8:**
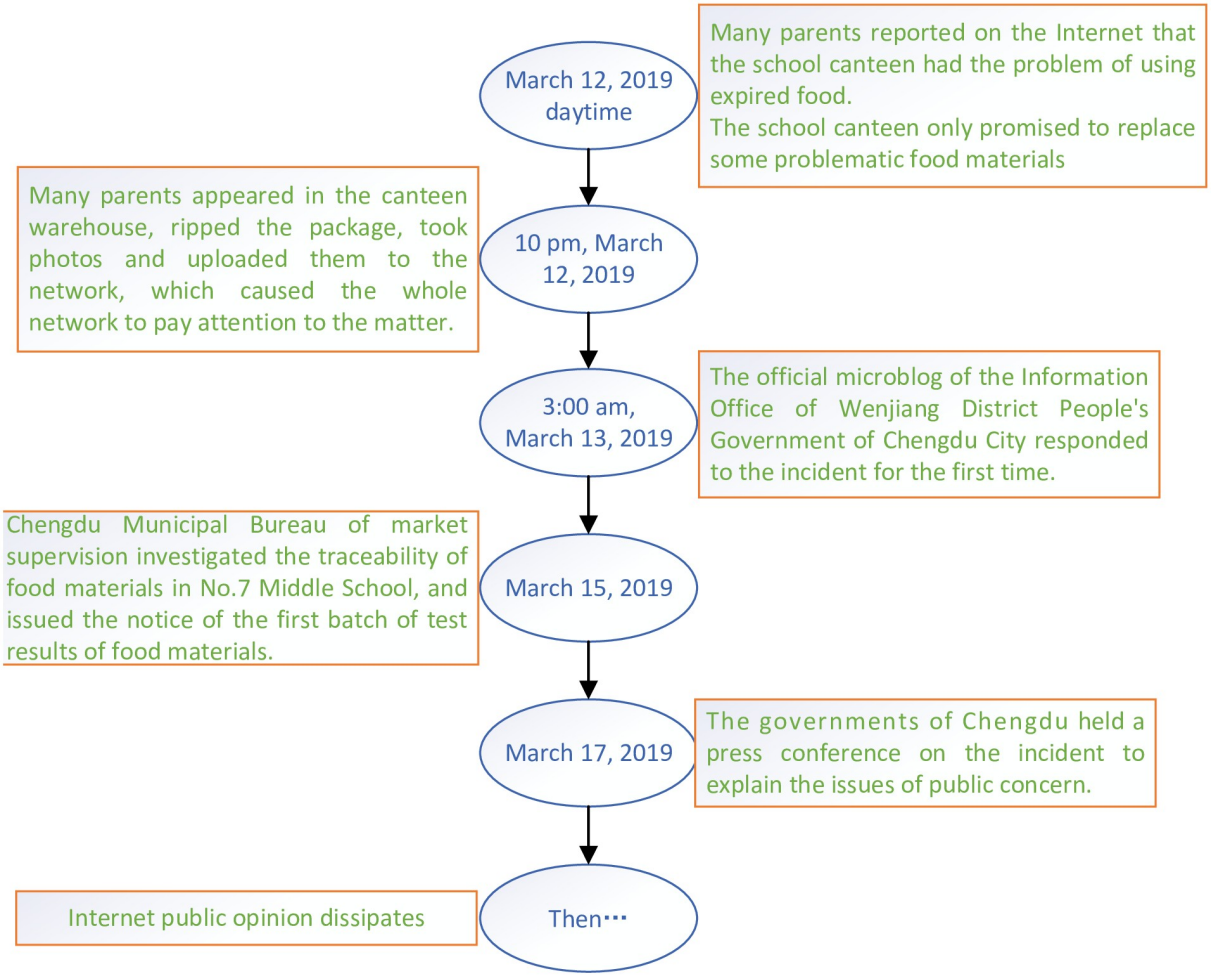
The process of a realistic case.

**Fig 9:**
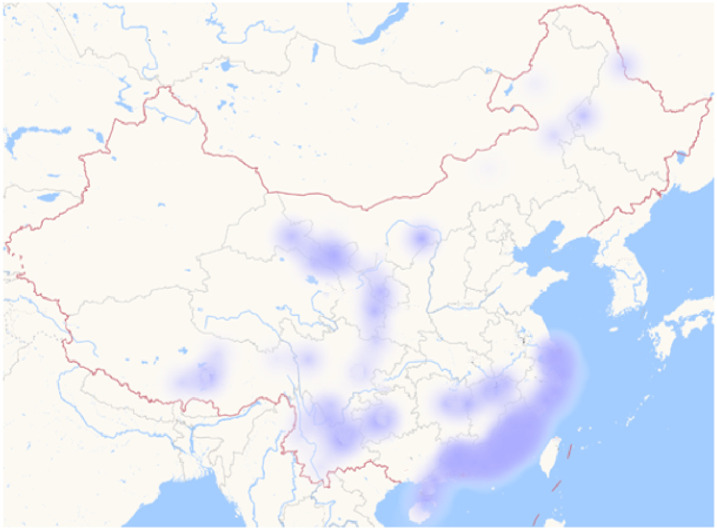
The process of a realistic case [25]. Republished from [https://unsplash.com/s/photos/china-map] under a CC BY license, with permission from [Unsplash], original copyright [original copyright 2020].

First, we obtained the distribution of network public opinion in the early stage of the event in the simulation model, which is shown in Fig 8. Then, due to the changes in the three key functions of relevant units, government departments, and internet users, there are undesirable links and invalid links, which lead to the outbreak of negative internet public opinion, the breeding of offline group events, and group polarization. The links are shown in Fig 9.

**Fig 10:**
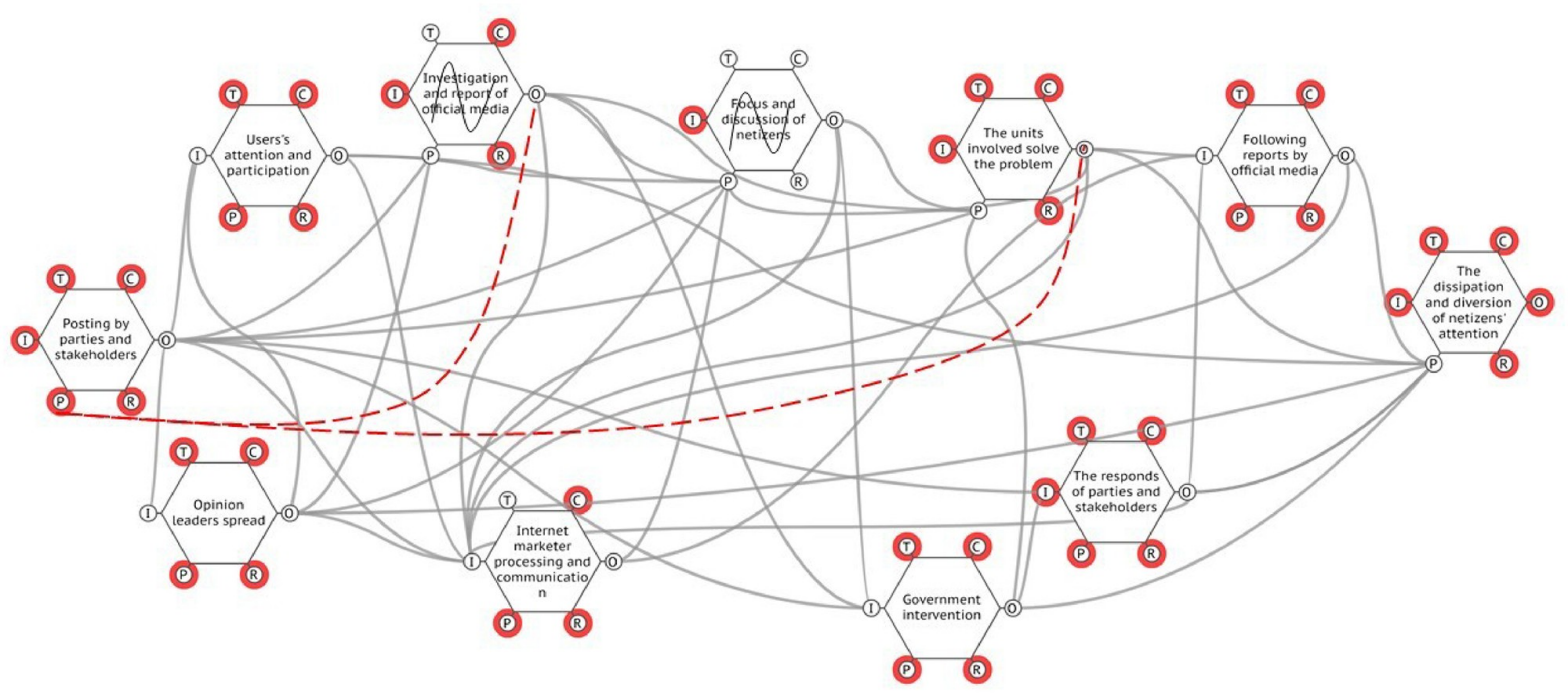
First simulation.

After using the simulation model to analyze the event, it was found that the development law of network public opinion is consistent with that in the model. Both experienced three periods of growth, climax, and decline. In Fig 10, each picture represents the distribution of public opinion at a certain moment.

Due to the lack of close links and cooperation among functional units, the cycle of public opinion dissipation is still very long. Therefore, to make the public opinion subside within a short time, the second and third simulations are carried out, based on the simulation model.

In the second simulation experiment, the researchers changed the parameters of key functional units, opinion leaders’ communication, investigation and report of official media, and government intervention, in the climax of public opinion, so that the functional units began to cooperate. In this case, public opinion rapidly declined since its climax.

In the third simulation experiment, the researchers changed the parameters of key functional units, opinion leaders’ communication, investigation and report of official media, and government intervention, in the generation stage of public opinion. In this case, the network public opinion did not appear in the climax stage and quickly dissipated. This did not cause large-scale public opinion events.

## 6 Conclusions future works and the shortcomings of the research

In the past, academic research on internet public opinion focused more on a single event or a cluster of events of similar subjects and topics and roughly summarized their patterns based on their characteristics, causes, and evolution. However, with the development of communication technology and the popularity of the internet, the relationships among the subsystems in society are no longer simple and linear, but includes the interaction of multiple factors such as people, technology, and organizations, which involve the complex changes of multiple subsystems in series or in parallel.

This paper considers online public opinion as a complex socio-technical dynamic system, and identifies and describes the effectiveness and role of each functional subject in the evolution of online public opinion. The paper constructs a functional network diagram of the evolution of online public opinion; attempts to identify the functional units that are prone to generating faults and trigger functional resonance from each function; explores how the complex factors in the evolution of online public opinion interact and couple from the functional resonance; clarifies the development logic of the evolution of online public opinion; and provides new research ideas for the prediction and governance of online public opinion.

Through the analysis of the online public opinion system using the FRAM, we derived 11 functions and functional network diagrams. Furthermore, we identified key functions that are prone to change during the evolution of online public opinion. In the face of a complex and ever-changing online public opinion system, online public opinion governance should firstly clarify the functions that each functional unit should perform. These include whether the government, the event-related institutions, official media and netizens are running their functions properly and monitor and prevent the generation of functional changes. They also address cutting off the coupling between functions when the functions change, to prevent changes in upstream functions from affecting downstream functions and avoiding the generation of functional resonance. In this way, the balance of the online public opinion system can be ensured and the generation of online public opinion risk accidents can be prevented.

The current research is insufficient in terms of practical applicability and ethics and the FRAM model based on deep learning in this study cannot fully cover all the details of the real environment. Furthermore, the practical adaptability of the model needs to be further tested and improved. Moreover, the ethical aspect of collecting the data of user accounts on social media for use in research is a major ethical problem.

In future research on public opinion governance, we ought to assess the theoretical model of functional resonance in the real public opinion environment, accept the reality test, and constantly revise the model. Second, we should integrate the emotional logic into the model, reflect the complexity and emergence of network public opinion, pay attention to the nodes that cause emotional resonance, and turn the focus of governance from content to emotion. Finally, we should integrate the background of digital humanities into it, and study the evolution and governance system of public opinion with the combination of big data and theory.

## Supporting information

S1 Data (RAR)

## References

[1] China Internet Network Information Center (CNNIC). (2020). The 46th China Internet Network Development State Statistical Report. Accessed: Sep.29, 2020. [Online]. Available from: http://www.cac.gov.cn/2020-09/29/c_1602939918747816.htm

[2] Kermack W O, McKendrick A G. Contributions to the mathematical theory of epidemics. II.-The problem of endemicity. Proceedings of the Royal Society of London. Series A, containing papers of a mathematical and physical character, 1932, 138(834): 55–83.

[3] Kitsak M, Gallos L K, Havlin S, et al. Identification of influential spreaders in complex networks. Nature physics, 2010, 6(11): 888–893. doi: 10.1038/nphys1746

[4] Elgazzar A S. Applications of small-world networks to some socio-economic systems. Physica A: Statistical Mechanics and its Applications, 2003, 324(1-2): 402–407. doi: 10.1016/S0378-4371(02)01956-8

[5] Tomassini M, Giacobini M, Darabos C. Evolution and dynamics of small-world cellular automata. Complex systems, 2005, 15(4): 261–284.

[6] Pȩkalski A. Ising model on a small world network. Physical Review E, 2001, 64(5): 057104. doi: 10.1103/PhysRevE.64.057104 11736145

[7] Bonzom V, Gurau R, Rivasseau V. The Ising model on random lattices in arbitrary dimensions. Physics Letters B, 2012, 711(1): 88–96. doi: 10.1016/j.physletb.2012.03.054

[8] Suchecki K, Eguíluz V M, San Miguel M. Voter model dynamics in complex networks: Role of dimensionality, disorder, and degree distribution. Physical Review E, 2005, 72(3): 036132. doi: 10.1103/PhysRevE.72.036132 16241540

[9] Li S T, Tsai F C. A fuzzy conceptualization model for text mining with application in opinion polarity classification. Knowledge-Based Systems, 2013, 39: 23–33. doi: 10.1016/j.knosys.2012.10.005

[10] Zhang H, Sekhari A, Ouzrout Y, et al. Jointly identifying opinion mining elements and fuzzy measurement of opinion intensity to analyze product features. Engineering Applications of Artificial Intelligence, 2016, 47: 122–139. doi: 10.1016/j.engappai.2015.06.007

[11] Kupennan M, Abramson G, Newman M, et al. Small world effect in an epidemiological model. The Structure and Dynamics of Networks. Princeton University Press, 2011: 489–492.

[12] Erik H. FRAM: the functional resonance analysis method: modelling complex socio-technical systems. CRC Press, 2017.

[13] Rosa L V, Haddad A N, de Carvalho P V R. Assessing risk in sustainable construction using the Functional Resonance Analysis Method (FRAM). Cognition, Technology & Work, 2015, 17(4): 559–573. doi: 10.1007/s10111-015-0337-z

[14] Li T, Raizen M G. Brownian motion at short time scales. Annalen der Physik, 2013, 525(4): 281–295. doi: 10.1002/andp.201200232

[15] Gammaitoni L, Hänggi P, Jung P, et al. Stochastic resonance. Reviews of modern physics, 1998, 70(1): 223. doi: 10.1103/RevModPhys.70.223 12779764

[16] McNamara B, Wiesenfeld K. Theory of stochastic resonance. Physical review A, 1989, 39(9): 4854. doi: 10.1103/PhysRevA.39.4854 9901841

[17] Patriarca R, Di Gravio G, Costantino F, et al. The Functional Resonance Analysis Method for a systemic risk based environmental auditing in a sinter plant: A semi-quantitative approach. Environmental Impact Assessment Review, 2017, 63: 72–86. doi: 10.1016/j.eiar.2016.12.002

[18] Chen H, Liu J, Li Y, et al. A two-stage dynamic undesirable data envelopment analysis model focused on media reports and the impact on energy and health efficiency. International journal of environmental research and public health, 2019, 16(9): 1535. doi: 10.3390/ijerph16091535 31052235 PMC6539354

[19] Wei-dong H, Qian W, Jie C. Tracing public opinion propagation and emotional evolution based on public emergencies in social networks. International Journal of Computers Communications & Control, 2018, 13(1): 129–142. doi: 10.15837/ijccc.2018.1.3176

[20] Falegnami A, Costantino F, Di Gravio G, et al. Unveil key functions in socio-technical systems: mapping FRAM into a multilayer network. Cognition, Technology & Work, 2020, 22(4): 877–899. doi: 10.1007/s10111-019-00612-0

[21] Riccardo P, Gianluca D P, Giulio D G, et al. FRAM for systemic accident analysis: a matrix representation of functional resonance. International Journal of Reliability, Quality and Safety Engineering, 2018, 25(01): 1850001. doi: 10.1142/S0218539318500018

[22] El Alaoui I, Gahi Y, Messoussi R, et al. A novel adaptable approach for sentiment analysis on big social data. Journal of Big Data, 2018, 5(1): 1–18. doi: 10.1186/s40537-018-0120-0

[23] Ergasheva M P. PUBLIC OPINION AND ITS ROLE IN THE SOCIAL LIFE OF SOCIETY. Theoretical & Applied Science, 2019 (10): 183–185. doi: 10.15863/TAS.2019.10.78.33

[24] Luo Q, Zhai X. “I will never go to Hong Kong again!” How the secondary crisis communication of “Occupy Central” on Weibo shifted to a tourism boycott. Tourism Management, 2017, 62: 159–172. doi: 10.1016/j.tourman.2017.04.007 32287749 PMC7125760

[25] The map available from: https://unsplash.com/s/photos/china-map

